# Effect of different thread configurations on hydrophilic implant stability. A split-mouth RCT

**DOI:** 10.1590/0103-6440202405632

**Published:** 2024-03-22

**Authors:** Pablo Pádua Barbosa, Vithor Xavier Resende de Oliveira, João Vitor Goulart, Rogério Margonar, Marcos Boaventura de Moura, Guilherme José Pimentel Lopes de Oliveira

**Affiliations:** 1 Dental School, Universidade Federal de Uberlândia-UFU, Uberlândia, Minas Gerais, Brazil.; 2 Dental School, Centro Universitário de Santa Fé do Sul- Unifunec, Santa Fé do Sul, São Paulo, Brazil.; 3 Department of Health Sciences, Implantology Post Graduation Course, Dental School, University Center of Araraquara(UNIARA), Araraquara, São Paulo, Brazil.

**Keywords:** implant design, stability, osseointegration

## Abstract

This split-mouth randomized controlled trial aimed to evaluate the primary and secondary stability of hybrid implants with different thread configurations and hydrophilic surfaces. Twenty patients with a partially edentulous maxilla were selected. These patients received two types of implants with the same hydrophilic surface: CTP group: Cylindrical-Tapered implant with perforating threads; CTH: Cylindrical-Tapered implant with hybrid threads configuration (perforating and condensing threads). The primary and secondary stability parameters were measured by insertion torque and resonance frequency analysis at the time of implant placement and 7, 28, 56, and 90 days after the surgical procedure. The paired t-test was used to compare the data on the implant's stability between the groups. The statistical analysis was performed with a confidence level set at 95%. It was found that the implants in the CTH group presented higher primary stability values ​​at the time of implant placement, due to the higher ISQ (63.61 ± 9.44 vs. 40.59 ±7.46) and insertion torque (36.92 ± 16.50 Ncm vs. 28.00 ± 14.40 Ncm), than the implants in the CTP group. The CTH group presented higher ISQ values ​​in all follow-up periods: 7 days (68.67 ± 7.60 vs. 41.55 ± 9.07), 28 days (68.61 ± 5.98 vs. 47.90 ±13.10), 56 days (74.09 ± 3.96 vs. 55.85 ± 13.18), and 90 days (75.45 ± 4.02 vs. 63.47 ± 6.92) after implant placement. Hybrid implants with perforating and condensing threads demonstrated greater stability than hybrid implants with only perforating threads.

## Resumo

O objetivo deste estudo controlado randomizado de boca dividida foi avaliar a estabilidade primária e secundária de implantes híbridos com diferentes configurações de rosca e superfícies hidrofílicas. Vinte pacientes com maxila parcialmente edêntula foram selecionados. Esses pacientes receberam dois tipos de implantes com a mesma superfície hidrofílica: Grupo CTP: Implante cilíndrico-cônico com roscas perfurantes; Grupo CTH: Implante cilíndrico-cônico com roscas perfurantes e condensadoras. Os parâmetros de estabilidade primária e secundária foram medidos por análise de torque de inserção e frequência de ressonância no momento da colocação do implante e 7, 28, 56 e 90 dias após o procedimento cirúrgico. O teste t-pareado foi utilizado para comparação dos dados de estabilidade entre os grupos. A análise estatística foi executada levando-se em consideração um nível de confiança de 95%. Constatou-se que os implantes do grupo CTH apresentaram maiores valores de estabilidade primária no momento da colocação do implante, devido ao maior ISQ (63.61 ± 9.44 vs. 40.59 ±7.46) e torque de inserção (36.92 ± 16.50 Ncm vs. 28.00 ± 14.40 Ncm), do que os implantes do grupo CTP. Implantes do grupo CTH apresentaram valores de ISQ superiores em todos os períodos de acompanhamento: 7 dias (68.67 ± 7.60 vs. 41.55 ± 9.07), 28 dias (68.61 ± 5.98 vs. 47.90 ±13.10), 56 dias (74.09 ± 3.96 vs. 55.85 ± 13.18) e 90 dias (75.45 ± 4.02 vs. 63.47 ± 6.92). Os implantes híbridos com roscas perfurantes e condensantes demonstraram maior estabilidade do que os implantes híbridos com apenas roscas perfurantes.

## Introduction

Dental implants have been used extensively in oral rehabilitation of all types of edentulism ^1^. However, despite the high survival and success rates, failures still occur, which may be associated with mechanical or biological factors that occur mainly in the first year of the implant function, especially at the critical moment for achieving osseointegration ^1^.

One controllable factor linked to success in osseointegration is the achievement of good primary stability, which makes secondary stability a more predictable event ^2^ ^,^ ^3^. Structural modifications in dental implants have been proposed to optimize the osseointegration process, and these modifications can be performed in the macrostructure or microstructure of the implants ^3^ ^,^ ^4^ ^,^ ^7^. Changes in the macrostructure more directly affect primary stability and the decision to establish immediate load application ^3^ ^,^ ^6^ ^,^ ^8^, while microstructural modifications are related to the acceleration of the conversion from primary to secondary stability due to biological stimuli in the osseointegration process ^5^ ^,^ ^9^ ^,^ ^10^ ^,^ ^11^.

Regarding the macrostructure of dental implants, previous studies have shown that tapered implants present higher primary stability than cylindrical implants ^8^ ^,^ ^12^, and this effect may also affect the acceleration of osseointegration ^8^. However, tapered implants can exacerbate the degree of primary stability in denser bones ^13^. Thus, implants with a hybrid structure; cylindrical in the coronal portion and tapered in the apical portion, have been proposed as an alternative to be used in any type of bone density ^7^ ^,^ ^14^, which could simplify the clinician's decision-making regarding the type of macrostructure to be used in different clinical conditions. Furthermore, modification in the shape of the implant threads has been proposed, to perforate and compress the surgical site to improve and control the primary stability of the hybrid implants ^7^. Thus, the objective of this clinical trial was to evaluate the primary and secondary stability of hybrid implants with different thread configurations. The null hypothesis of this study is that the different body and thread configurations of the implants will not influence the primary and secondary stability of the dental implants.

## Material and methods

### Ethical considerations and patient selection

The ethical committee of the University of Santa Fé do Sul, Brazil approved this split-mouth randomized controlled clinical trial (CAAE: 37995520.7.1001.5428). The study protocol was registered in the Brazilian Registry of Clinical Trials (ReBEC - U1111-1263-9721). This study followed the ethical precepts set out in the Declaration of Helsinki and was conducted in compliance with the CONSORT guidelines.

The patients were treated, and the data were collected at the post-graduation clinics of the University of Santa Fé do Sul between May/2021 and January/2022. One operator (PPB) enrolled all the patients. Twenty patients undergoing installation of at least one pair of implants participated in this study. The patients were selected according to the following inclusion criteria: 1) Aged between 18 and 60 years; 2) Requiring single or multiple, bilateral or unilateral rehabilitation with osseointegrated implants; 3) Sufficient bone available for installation of a conventional size implant; 5) Tooth extraction performed at least 6 months before the placement of the implant; 6) Good systemic health.

Patients with the following characteristics were excluded from this study: 1) Smokers; 2) Uncompensated diabetics; 3) Patients who are chronic users of medications (e.g., bisphosphonates, immunosuppressants, anti-inflammatory drugs) or with pathologies that alter bone metabolism; 4) Patients who chronically use anti-inflammatory drugs and antibiotics; 5) Bruxism; 6) Chemical dependency; 7) Pregnant or who want to get pregnant in the next year; 8) History of radiotherapy treatment in the head and neck region.

To calculate the sample size, the stability of the implants measured by resonance frequency analysis was used as the primary variable. A study comparing the stability of implants with different macrostructures (Cylindrical vs. Tapered) demonstrated an expected standard deviation for this analysis of 5.19 ^8^. Considering a minimum clinically relevant difference for the ISQ of 5 points and setting the β power at 0.90 and type I error at 0.05, it was determined that at least 15 patients would be necessary to carry out this study.

### Surgical procedure and groups

**Figure 1:**
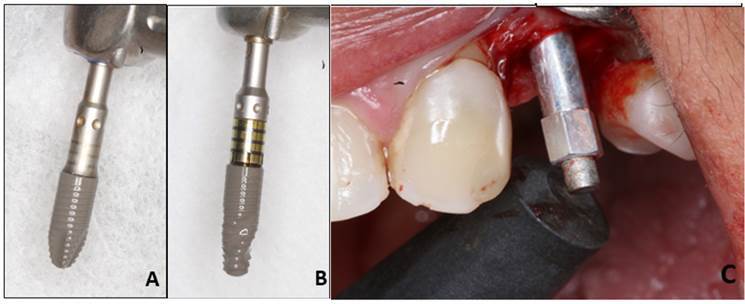
A) CTH implant; B) CTP implant; C) Resonance frequency analysis

After performing the local anesthesia technique, a full-thickness mucoperiosteal flap was opened to expose the maxillary ridge. The milling procedure was carried out under abundant irrigation with saline solution and by the implant manufacturer's recommendations. The implant sites were randomly allocated to receive one of the two implant types. The implants in the CTP group were cylindrical in the middle and coronal portion and tapered in the apical portion with perforating threads (Titamax EX®, Cone Morse, Neodent, Curitiba, Brazil) and the implants in the CTH group were cylindrical in the coronal portion and conical in the apical and middle portion with hybrid threads configuration (perforating and condensing threads) (Helix®, Grand Morse, Neodent, Curitiba, Brazil) (Figure 1) ^7^. The implants had a diameter of 3.75 mm and a length of 9 mm (CTP) or 10 mm (CTH), presented morse taper connection, the same hydrophilic surface (Acqua, Neodent, Curitiba, Brazil), and were installed 2mm subcrestally. Randomization was performed by applying a randomization table at the time of perforation of the site for implant placement (random.org) (GJO). The patients were blinded regarding the location of the different types of implants.

After insertion of the implants, the surgical site was sutured with 5.0 nylon threads (Ethicon, Johnson & Johnson, Brazil). Post-operative care included oral application of amoxicillin (500mg) for 7 days, nimesulide (100mg) for 5 days, and sodium dipyrone (500mg) for 3 days. Additionally, 0.12% chlorhexidine gluconate-based mouthwash was prescribed for 14 days. The sutures were removed after 7 days. The healing abutment (Neodent, Curitiba, Brazil) was installed and maintained for 90 days after implant placement when the implants were submitted to prosthetic loading when CM Abutments (CTP Group; Neodent) and GM Exact Abutments (CTH group; Neodent) were inserted (PPB). One operator (PPB) performed all the surgeries while two blinded evaluators (JVG; VXO) collected the clinical data.

### Analysis of stability of the dental implants

**Figure 2:**
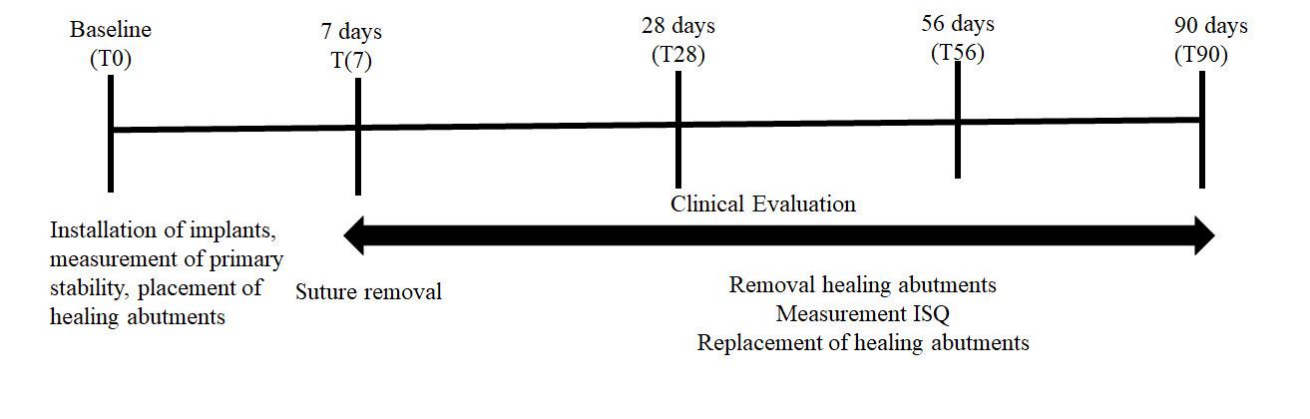
Flowchart showing the study design

At the time of implant placement, primary stability was measured through insertion torque and resonance frequency analysis using the Osstell® device (Osstell AB, Göteborg, Sweden). The system includes the use of a specific SmartPeg for each implant, which is fixed to the implant by an integrated screw. The SmartPeg is then excited by a magnetic impulse from the measuring probe of the portable instrument and the implant stability coefficient (ISQ) is calculated. The results are displayed on the instrument, varying on a scale from 1 to 100, in that the higher the ISQ number, the greater the stability of the implant. Stability measurements were obtained on four faces of each implant (buccal, palatal, distal, and mesial) and the mean of the results was considered the stability value of each implant. The ISQ evaluation was measured again 7, 28, 56, and 90 days after implant placement (Figure 2) (JVG; VXO).

### Statistical analysis

GraphPad Prism 6 software (San Diego, CA, USA) was used to perform the statistical analysis of this study. Numerical data from stability analyses demonstrated normal distribution according to the Shapiro-Wilk test. The comparisons between the groups of implants in each follow-up period were evaluated using the paired t-test. Longitudinal data within each group of implants were evaluated using repeated measurements ANOVA complemented by the post-hoc Tukey test. All tests were applied with a confidence level set at 95%.

## Results

**Figure 3:**
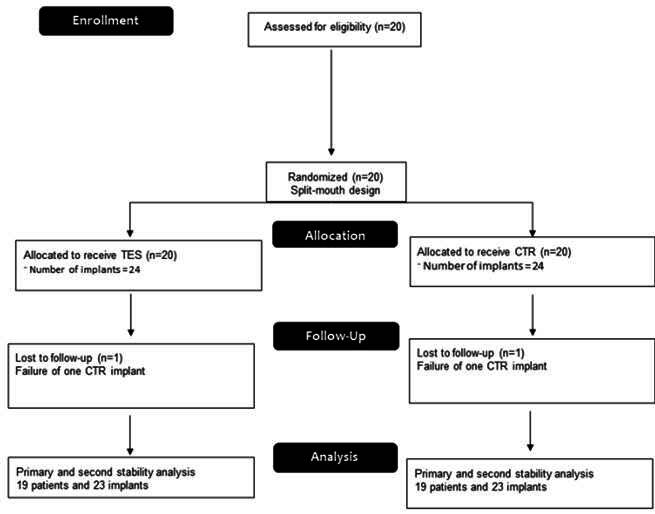
Flow diagram of the study.

Forty-eight implants were installed in the maxillary region in 20 patients (6 men and 14 women; aged between 22-60 years). Patients received 2 (16 patients) or 4 implants (4 patients), with 24 implants placed in the CTP group and 24 implants placed in the CTH group. During the evaluation period, one implant in the CTP group was lost, which generated a survival rate of 95.83% for implants in the CTP group and a 100% survival rate in the CTH group. The lost implant was replaced by another implant from the CTP group, but the patient was removed from the follow-ups. Thus, 46 implants placed in 19 patients were included in the final evaluation (Figure 3, Table 1). The quality of the bone site where the implants were placed presented no differences between the groups (Table 1).

**Table 1:** Baseline clinical characteristics for each group

Sites/Groups	CTP	CTH
Sites		
Canine	1	1
First premolar	6	8
Second premolar	7	6
First molar	5	4
Second molar	4	4
Total	23	23
Bone quality		
I	-	-
II	3	3
III	5	6
IV	15	14
Total	23	23

The data provided by the implant’s stability analysis were described as mean and standard deviations. The implants in the CTH group presented higher insertion torques than the implants in the CTP group (36.92 ± 16.50 Ncm vs. 28.00 ± 14.40 Ncm) (p<0.05). In addition, implants in the CTH group had higher ISQ values ​​than implants in the CTP group at baseline (63.61 ± 9.44 vs. 40.59 ±7.46), 7 days (68.67 ± 7.60 vs. 41.55 ± 9.07), 28 days (68.61 ± 5.98 vs. 47.90 ±13.10), 56 days (74.09 ± 3.96 vs. 55.85 ± 13.18), and 90 days (75.45 ± 4.02 vs. 63.47 ± 6.92) after implant placement (p<0.05) (Table 2). The implant stability increased in both types of implants over the follow-up period (p<0.05) (Table 2).

**Table 2:** Mean, standard deviation, and the 95% confidence interval of the ISQ values for the CTP and CTH groups. *p<0.05 - Higher ISQ than the CTP groups. Paired t-test.

Period/Group	CTP	CTH
Baseline	40.59 ± 7.46 (37.44 - 43.74)	63.61 ± 9.44 (59.62 - 67.60)^*^
7 d	41.55 ± 9.07 (36.88 - 46.21)	68.67 ± 7.60 (65.01 - 72.34)^*^
28 d	47.90 ± 13.10 (42.09 - 53.71)	68.61 ± 5.98 (66.02 - 71.19)^*^
56 d	55.85 ± 13.18 (50.01 - 61.70)	74.09 ± 3.96 (72.38 - 75-81)^*^
90 d	63.47 ± 6.92 (60.13 - 66.81)	75.45 ± 4.02 (73.57 - 77.33)^*^

## Discussion

In the current study, implants with a hybrid macrostructure, associating perforating and condensing threads, presented greater primary stability and acceleration of the conversion from primary to secondary stability compared to hybrid implants with only perforating threads. These findings suggest that implants in the CTH group may increase predictability in establishing osseointegration at its most critical moment, and make protocols for early implant loading safer. In fact, CTH implants also had a higher survival rate than CTP implants.

The success of dental implants is directly influenced by the level of primary stability after implant placement ^15^, and the implant macrostructure plays an important role in achieving primary stability ^7^ ^,^ ^12^. The shape of implants and the thread configurations influence this parameter, as demonstrated in previous studies in which tapered implants presented greater stability than cylindrical implants ^8^ ^,^ ^12^ ^,^ ^15^. The implants used in the current study have a hybrid macrostructure in which there is an association of a cylindrical shape in the coronal portion and a tapered shape in the lower portion with a double-threaded design, and this type of implant macrostructure has also been shown to present superior stability to cylindrical implants with single-threaded design ^7^ ^,^ ^16^. Taking into account the data obtained on primary stability in this study, both implants presented adequate values of insertion torque, which demonstrates the effectiveness of this type of implant format in obtaining locking even in low-density bone such as in the posterior region of the jaw.

Regarding the shape of the implant threads, studies have shown that different thread configurations present better results depending on the type of bone where the implants are installed ^17^ ^,^ ^18^. Triangular or sharp threads reduce bone resistance during implant insertion by inducing cuts in the bone structure, which facilitate implant placement in high-density bone ^19^ ^,^ ^20^. On the other hand, the condensing square threads compress the bony trabeculae and increase locking in bone with low density ^17^. The CTH implant presents the association of these two types of threads, the perforating triangular threads are located in the apical portion, while the square condensing threads are located at the coronal portion and this characteristic may explain the superiority in the primary stability of the CTH implants compared with the CTP implants.

Primary stability is a good predictor of the osseointegration process, and this requirement has been used as a determining factor for the best time to apply prosthetic loading ^1^ ^,^ ^3^, and the only implant lost during in this study was one implant of the CTP group that presented insertion torque lower than 20 Ncm and ISQ lower than 40. In fact, implants in the CTH group showed higher levels of secondary stability throughout the study, which corroborates other studies that emphasize the importance of primary stability in achieving faster and more predictable osseointegration ^21^. The greater stability obtained in CTH implants likely makes this type of implant safer for immediate or early loading protocols in low-density bone, but this hypothesis needs to be tested in the future.

Another important aspect of the evolution of the osseointegration process is the characteristics of the implant surfaces ^14^. In this study, both implants had a hydrophilic surface, with double acid etching and sandblasting before being kept in isotonic solution. Histological analysis of bone-implant contact has shown that this surface accelerates the osseointegration process in preclinical (4, 9) and clinical ^5^ studies, compared to implant surfaces with similar surface treatment but without the same level of high wettability. This acceleration in osseointegration is related to increased osteogenesis, which is increased on this type of surface due to the greater adhesion of undifferentiated mesenchymal cells ^22^ and stimulation in the differentiation and activity of osteoblasts, which increase bone formation rates ^23^. However, the clinical superiority of the hydrophilic surface in primary stability and its conversion to secondary stability has not been confirmed in clinical studies ^14^ ^,^ ^24^. It is possible that in clinical situations where implants achieve good primary stability the importance of the type of surface used is reduced. Although the implants in the current study presented the same surface, the transition process from primary to secondary stability occurred differently due to the different macrostructural characteristics of the tested implants.

This study has some limitations that must be taken into account when interpreting the obtained findings. The implants were not subjected to occlusal loading, and it is known that loading can stimulate the acceleration of the osseointegration process ^25^. The impact of these implants' macrostructures on different bone quality and in patients with systemic conditions that alter bone remodeling requires more investigation. In addition, long-term assessments to determine whether these different macrostructures significantly affect implant success and survival are also needed. Finally, the lengths of the implants used were different (CTH - 10 mm vs. CTP - 9 mm), but this characteristic does not seem to significantly affect the stability of the implants compared with the implant’s diameter ^6^.

## Conclusion

Hydrophilic hybrid implants with perforating and condensing threads demonstrate greater stability than hybrid implants with only perforating threads. Then, the null hypothesis of this study was rejected.
